# A Measure of Perceived Chronic Social Adversity: Development and Validation

**DOI:** 10.3389/fpsyg.2017.02168

**Published:** 2017-12-12

**Authors:** Jingqiu Zhang, Cody Ding, Yunglung Tang, Chunyu Zhang, Dong Yang

**Affiliations:** ^1^School of Psychology, Southwest University, Chongqing, China; ^2^Key Laboratory of Cognition and Personality, Ministry of Education, Southwest University, Chongqing, China; ^3^Department of Educational Psychology, Research, and Evaluation, University of Missouri—St. Louis, St. Louis, MO, United States; ^4^School of Psychology, Shaanxi Normal University, Xi'an, China

**Keywords:** perceived chronic social adversity, measurement, reliability, construct validity, concurrent validity, psychological difficulty

## Abstract

The goal of this study was to develop a measure that assesses negative daily social encounters. Specifically, we examined the concept of perceived chronic social adversity and its assessment, the Perceived Chronic Social Adversity Questionnaire (PCSAQ). The PCSAQ focused on the subjective processing of daily social experiences. Psychometric properties were examined within two non-clinical samples (*N* = 331 and *N* = 390) and one clinical sample (*N* = 86). Exploratory and confirmatory factor analyses supported a three-factor model of the PCSAQ, which corresponds to three types of daily social stressors. The final 28-item PCSAQ was shown to be internally consistent, and to have good construct validity in terms of factor structure and group differences. It was also shown to have good concurrent validity in terms of association with outcome variables (sense of control, happiness, and mood and anxiety symptoms). Perceived chronic social adversity was also shown to be correlated with PTSD severity. Taken together, these findings suggest that the PCSAQ is a reliable, valid, and useful measure that can be used to assess negative social and clinical aspects of personal experiences. This study is an important exploratory step in improving our understanding of the relationship between the cumulative effect of negative social encounters and psychological difficulty.

## Introduction

In daily lives, we often observe that when someone experienced a series of negative daily social encounters (such as deception, unfair treatment, emotional blackmail, discrimination, bullying, or unemployment), they could experience psychological distress, including re-experience, hypervigilance, anxiety, or depression. Although they are not life-and-safety threatening events, cumulative exposure to these negative daily encounters might lead to difficulties, especially when these aversive situations are unavoidable over a longer period of time or when they occur repeatedly and accumulatively. The prolonged stress could lead to wear-and-tear on the body and mind (McEwen, 2004; Charles et al., 2013), and may in turn affect one's sense of control and ability to cope with stress. For example, according to Polani (2004) and Tafet and Smolovich (2004), anxiety and depression probably represent different temporal phases of response to persistent, unresolved stressors. McEwen (2001) suggested that repeated stress can cause allostatic load, which is referring to the cost to the body of adaptation to adverse conditions. Caston and Frazier (2013) found that greater distress was related to perceived less present and future control over an event that was actually more controllable.

One question is whether cumulative exposure to negative daily encounters could be perceived as chronical adversities and be related to traumatic reactions from the perspective of individuals involved? From a constructionist standpoint, a personal construct system is subjectively evolved in order to make sense of the world. When his/her assumptive world is fractured, he/she may experience a traumatic reaction (e.g., McCann et al., 1988; Janoff-Bulman, 1992; Butt and Parton, 2005). Similarly, Craparo (2016) argued that how the individual perceives the event is more important than whether the event itself is objectively considered as a trauma.

Based on these studies and our own observations, we speculate that if negative daily social encounters occur repeatedly, continuously, or accumulatively, these encounters may be perceived by individuals as very stressful and even potentially overwhelming and likely to lead to physical or mental difficulties such as traumatic feeling or reactions, regardless of their true nature. The goal of this study was to develop a measure of perceived chronic social adversity that may result from our daily social encounters. Such a measure can help assess whether social daily encounters may appear negative to individuals and how much these encounters disturb them.

### Conceptual framework of perceived chronic social adversity

In our conceptualization of perceived chronic social adversity, we consider perceived chronic social adversity as a series of emotionally directed and stressful events that people personally perceived as stressful and even potentially overwhelming because these events occur repeatedly, continuously, or accumulatively during social interactions and social competition. It can be characterized as “social,” “emotionally directed,” “chronic,” and “perceived.” “Social” indicates a focus on stressful events involving either interpersonal relationships or social competition. “Emotionally directed” describes the way in which emotions are affected by events that do not cause direct physical harm but rather are experienced as injustice, inequality, disrespect, rejection, discrimination, bullying, and so on. “Chronic” illustrates the long duration or recurrence of such experienced events causing emotional distress. The cumulative effect of minor emotionally distressed stressors, particularly when they are continuous, can eventually lead to major mental suffering in relation to values, status, or self-worth. “Perceived” describes the subjective or unique way in which individuals evaluate these experiences. The same event could be evaluated as very stressful or even potentially overwhelming by one person but not another, depending on previous personal experiences and the way in which individuals experience or perceive negative events.

Since there are possibly many daily social encounters, we focus on three types of social events that may be most likely to be perceived as aversive: obvious or obscure social exclusion or alienation, being overly controlled, and weakness in social competition. We restricted perceived chronic social adversity to these three types of events because first these social events are common in social situations. Second, from the perspective of evolutionary psychology, individuals need to mobilize personal resources and integrate themselves into certain social networks to cope with environmental challenges and compete with other individuals for limited resources (Campbell et al., 1986; Kanazawa and Savage, 2009). Negative social interaction and weakness in social competition can reduce the amount of social resources that individuals may obtain, especially when they happen repeatedly or continuously. Third, from the perspective of existentialism, positive interpersonal relationship and personal excellence are powerful defense mechanisms to obtain a sense of security and relieve individual anxiety about survival (Yalom, 1980). When these two aims are threatened, individuals are more likely to feel defenseless in the face of overwhelming anxiety.

For the first type of event of “obvious or obscure social exclusion or alienation,” it is represented, either perceived or real, by deliberate abandonment, rejection, exclusion, unfair treatment, discrimination, disregard, suspicion, deception, or devaluation. Unfair treatment is included because it represents situations in which a person is not valued or respected by other group members and may thus generally be perceived as a threat to social inclusion (Lind and Tyler, 1988). Relational devaluation is incorporated because it signals increased probability of ultimate exclusion (MacDonald and Leary, 2005). Similarly, we believe that discrimination, disregard, suspicion, and deception symbolize insincere treatment or lack of respect leading to exclusion or alienation.

For the second type of event of “being overly controlled,” it could be in a seemingly intimate relationship or obviously unequal relationship. The first example that comes to mind is the relationship involving authoritarian parents (Baumrind, 1967). These parents usually expect their children to follow instructions without question or discussion (Sharma et al., 2011) and attempt to intrude on children's psychological and emotional development (Ballash et al., 2006). Overcontrol also exists in relationships with spouses, peers, friends, colleagues, or supervisors. Emotional blackmail is a common form of overcontrol in these relationships. Forward and Frazier (1998, pp. x-xii) stated, “emotional blackmail is a powerful form of manipulation in which people close to us threaten, either directly or indirectly, to punish us for not doing what they want.” Knowing the victim's vulnerabilities or deepest secrets, blackmailers blur victims' clarity of judgment by intensifying their fear, obligation, and guilt.

For the third type of event of “weakness in social competition,” it refers to disadvantage, powerlessness, or lack of voices during social competition for opportunities of learning, job and status, all of which refer to social resources. Unlike the first two types of event, which happen during direct social interactions, this third type is not directly caused by others' negative responses but is ascribed to the blockage of “unidirectional drive upward” during social comparison. According to Festinger (1954), this is a basic drive that generally causes individuals to strive to do better and be more capable relative to comparative targets. Through social comparison, people attempt to protect and maintain their superiority in various contexts such as daily social situations or organizational settings (Garcia et al., 2013). Weakness in such social completion may lead to perceived unfairness, disrespect, inferiority, low self-esteem or worthlessness.

Therefore, we attempted to develop the Perceived Chronic Social Adversity Questionnaire (the PCSAQ) to systematically assess some common and major stressors in daily social life described above. Unlike existing measures of trauma/stress history, such as Post-traumatic Stress Diagnostic Scale (Foa, 1995), the life stressor checklist (Norris and Hamblen, 2004), the life event checklist (Weathers et al., 2013), the Traumatic Life Events Questionnaire (Kubany et al., 2000), and the Trauma History Screen (Carlson et al., 2011), the PCSAQ assess a series of emotionally-directed social events that could possibly be perceived by individuals as stressful and even potentially overwhelming. Such measure may help us to better understand the pathway to person's inner world from a longitudinal perspective. That is, assessment of perceived chronic social adversity may provide us with a better picture of the relationship between perceived social stressful events and psychological functioning. This step may be useful for individuals to receive timely treatment or for researchers to identify resiliency factors that may mitigate its negative effects. In the following sections, we described the procedures and studies used to develop the PCSAQ.

### Development of the PCSAQ

#### Preliminary item construction

Following the commonly used procedure for instrument development, we first used theoretically driven thematic facets to guide our item development. A comprehensive review of the literature on trauma or stressors was conducted to ensure that the potential item pool would reflect the breadth of the theorized perceived chronic social adversity content. Secondly, the trauma/stressor-related measures that were either completely or partially in accordance with our definition of perceived chronic social adversity were examined to obtain and modify the items suitable for the PCSAQ (see Table 1 for the list of measures reviewed).

**Table 1:** The trauma/stressor-related measures and their items suitable for the PCSAQ.

**Measures reviewed**	**Sample items**
The Brief Betrayal-Trauma Survey (BBTS; Goldberg and Freyd, 2006)	You were emotionally or psychologically mistreated over a significant period of time by someone with whom you were very close (such as a parent or lover).
The Ostracism Experience Scale for Adolescents (OESA; Gilman et al., 2013)	Treat me as if I am invisible. Invite me to join them for weekend activities, hobbies, or events (reverse coded).
Psychological Control scale -Youth self-Report (PCS-YSR; Barber, 1996)	Always trying to change how I feel or think about things. Brings up past mistakes when she (he) criticizes me.
USC Parental Overcontrol Scale (USC-POS; Borelli et al., 2015)	I am less friendly when my child doesn't see things my way. There are lots of ways that I'd like to change my child.
Questionnaires of Employees' Perception of Emotional Blackmail (EPEB; Chen, 2009)	Others use threaten language to make a demand. Others do their best to make me feel guilty.
The Internal-External Entrapment Scale (IEE; Gilbert and Allan, 1998)	I would like to get away from other more powerful people in my life. I can see no way out of my current situation.
The Defeat Scale (SDS; Gilbert and Allan, 1998)	I feel I have lost important battles in life. I feel that I am basically a winner (reverse coded).

The primary theoretical basis for selecting events was as follows. First, social, emotionally directed, chronic, and perceived valence are all necessary (but not sufficient) characteristics for an event to be incorporated into the PCSAQ; second, the event fell into one of three domains of perceived chronic social adversity as defined above. From this broad literature review and the existing related measures, an initial pool of 48 items were developed. The questionnaires items were originally developed in English and subsequently translated into Chinese by a linguist bilingual in Mandarin and English. We followed Brislin's (1980) back-translation procedure by another bilingual linguist to ensure the equivalence of the measures in the Chinese and English versions. Thirdly, we invited a focus group of 30 psychology postgraduates (18 masters and 12 doctoral students) to provide feedback concerning the items. A semi-open questionnaire was administered to collect information regarding item content, wording, and other possible items for inclusion. Based on this result, problematic items were rephrased or removed from the item pool, which resulted in a total of 33 items. Finally, to ensure that the items were representative of the three dimensions of perceived chronic social adversity, they were subsequently reviewed by four experts in the fields of trauma and stress. Based on this review, 31 items were kept for the subsequent analyses. All study procedures were approved by the Research Ethics Committee of the Faculty of Psychology, Southwest University, Chongqing (ID: 201746).

## Study 1: exploratory factor analysis

The aim of Study 1 was to refine the initial pool of 31 items using exploratory factor analysis (EFA). The results of this study were then subjected to validation in Studies 2 and 3.

### Methods

#### Participants

The sample consisted of 331 participants (23.9% undergraduates, 15.7% vocational college students, 28.4% soldiers, 29.3% adults from the community, and 2.7% other; 31.5% female and 68.5% male). The mean age was 24.72 years (*SD* = 8.36) for the entire sample, 20.23 (*SD* = 1.35) for the undergraduates, 18.66 (*SD* = 0.82) for vocational college students, 21.71 (*SD* = 2.83) for soldiers, and 34.55 (*SD* = 9.12) for adults from the community. Thirty-two percent of the participants (adults from the community and other) completed the questionnaire online, whereas students and soldiers, recruited from universities, vocational colleges and military bases in Chongqing and Chengdu, completed the pen-and-paper version in group settings. Online and paper completion have been found to have no substantial impact on cognitive processing of questionnaire items (Hardre et al., 2007; Davidov and Depner, 2011). We used the data from the participants who completed at least 95% of the items.

#### Procedure

All participants provided their written informed consent before they completed the questionnaires. Respondents completed the 31-item version of the PCSAQ. For each item, they first rated the extent to which the statement was in accordance with their experience on a 5-point response scale (i.e., rating of experience) from 1 to 5 (completely disagree, slightly agree, moderately agree, very agree, completely agree). If respondents endorsed the response options other than 1 (i.e., completely disagree), respondents were asked to further indicate how much that experience bothered them on a 4-point response scale (i.e., rating of disturbance) from 1 to 4 (not at all, slightly, obviously, extremely). Thus, PCSAQ items had two types of total scores: score on experience (PCSAQ-E) and score on disturbance (PCSAQ-D). For PCSAQ-D, all items were automatically scored as one when people response one to the first question (rating of experiences). The PCSAQ also included an additional open item inquiring about any other obviously or extremely disturbing daily social experiences not captured by the 31 items.

A unique feature of the PCSAQ is that it does not inquire how many times a certain type of stressor occurred. Rather, respondents need to report the extent to which they perceive that an event seems to always occur. We used this survey design because daily social events could be either continuous or recurring; thus, frequency estimates may be difficult to report accurately. Additionally, respondents' abilities to provide accurate frequency estimates for events that were years or decades in the past are likely to be poor. Furthermore, the actual focus of the PCSAQ is not objective facts such as natural disasters or accidents but the subjective processing of daily social experiences. It is not important how many times respondents actually experience social events or what the facts are. If respondents feel that they experience something repeatedly or continuously (chronic experience), they are more likely to perceive this event as “always” occurring. Thus, we expect respondents who report “always” may be at higher risk of mental disturbance. For example, if an individual completely agrees with the statement “I am always treated unfairly,” on the 5-point Likert-type scale, he/she will be more likely to experience perceived chronic social adversity.

#### Data analysis

To explore conceptual similarity and the coexistence of seemingly different social daily experiences, we conduct EFA and reliability analyses based on score of experience instead of score of disturbance since experience and disturbance are two aspects of perceived chronic social adversity rather than two distinct latent factors. We began the data analyses by using EFA to examine the factor structure of the 31-item PCSAQ-E. Factors were extracted based on a correlation matrix using the principal axis factoring method. Factors were rotated using the direct oblimin rotation (delta = 0). The number of factors was based on the results of the parallel analysis (Horn, 1965). The internal reliability of the total questionnaire and subscales was also estimated. All the analyses were conducted using SPSS 20.0.

### Results and discussion

For EFA, the Kaiser-Meyer-Olkin test of sampling adequacy was 0.879. The results of the parallel analysis suggested three factors, which was consistent with our expectations. In selecting items, we used the magnitude of factor loadings, face validity, and conceptual consideration to guide our choices. According to Tabachnick and Fidell (2007), an item with a factor loading less than 0.32 should be deleted because less than 10% of its variance can be explained by common factors. Consequently, original Item 2 (Always being betrayed by my lover or spouse) and original Item 31 (My business always fails) were deleted from the item pool. In addition, items with high cross loading leading to a difference under 0.20 between loadings on two factors were removed; thus original Item 12 (My private information always spread) was deleted from the item pool. Therefore, the final questionnaire consisted of 28 items. Factor 1 (overcontrol, OC) was composed of 13 items, with a Cronbach's alpha of 0.88; Factor 2 (social exclusion, SE) was composed of 10 items, with a Cronbach's alpha of 0.83; and Factor 3 (weakness in social competition, WISC) was composed of 5 items, with a Cronbach's alpha of 0.65. The internal reliability of the total questionnaire was 0.90. Table 2 shows the rotated factor pattern matrix of the final version. The inter-factor correlation for factors 1 and 2 was 0.48, for factors 2 and 3 was 0.32, and for factors 1 and 3 was 0.20. The items of the final Chinese form are in Appendix.

**Table 2:** Factor structure loadings for the 28 PCSAQ items.

**Item number**	**Factor 1**	**Factor 2**	**Factor 3**
22. Someone always reminds me or implies that if I really care about him (her), I would not do anything that bothers him (her).	0.69		
12. Someone does not allow me to query concerning his or her opinions and behaviors.	0.68		
15. Someone is always trying to change the way I do things.	0.66		
19. Someone forces me to meet her (his) demands by hurting or threatening to hurt me.	0.60		
17. Someone always brings up my past mistakes when she (he) argues with me.	0.60		
11. Somebody is always trying to change my feelings and thoughts.	0.59		
23. Someone lets me know in some way that I should be responsible for his or her distress, and only I can bring him (her) out of the predicament.	0.56		
21. Someone always reminds me or implies that he (she) has done a lot or made so many sacrifices for me.	0.55		
14. Someone arbitrarily makes most decisions for me.	0.54		
20. Someone will deprive me of what I care about if I don't comply with her (his) requirements.	0.53		
16. Someone always blames me for his/her or someone else's problems.	0.49		
13. Someone is always trying to restrict my freedom to speak.	0.47		
18. In order to force me to meet her (his) requirements, someone always hurts or threatens to hurt herself (himself) on purpose.	0.44		
8. Always being derogated.		0.65	
3. Always being ignored or left out.		0.62	
9. Always being slandered.		0.59	
6. Always being rejected.		0.59	
5. Always being excluded or isolated.		0.57	
2. Always being unfairly treated.		0.53	
10. Always being doubted or distrusted.		0.53	
7. Always being laughed at, teased, and/or humiliated.		0.53	
4. Always being deceived.		0.41	
1. Always being abandoned.		0.37	
27. It's always hard or impossible for me to fulfill a task.			0.60
24. I always underperform at work or in school.			0.54
25. I always fail in applying for schools or jobs.			0.53
28. I always fail in competition.			0.43
26. I am always dismissed.			0.33

*This table is based on the final version of the PCSAQ. Only loadings above 0.32 are presented in the table*.

An important point needs to be noted with respect to factor structure. Although the PCSAQ explores daily social experiences, which are not defined in terms of objective dimensions, their meaning is dependent on the way events are experienced by individuals. Factor analysis can contribute to explorations of similarity and the coexistence of seemingly different social daily experiences. However, it is necessary to distinguish the PCSAQ from questionnaires that assess constructs such as depression or anxiety. For example, psychological or behavioral symptoms of the same type tend to cluster together and form a factor; however, this may not be the case for various subjective experiences. Subjective experiences involve the individualized processing of objective experiences; thus, two individuals may evaluate the same experience in very different ways. This may be one reason for the relatively low internal consistency of the WISC factor and the relative low factor loadings of Items 1 and 28.

## Study 2: confirmatory factor analysis and validity

The aim of Study 2 was to replicate the factor structure of the SE, OC, and WISC developed in Study 1 using the final version of the PCSAQ, and to examine the construct and concurrent validity of the PCSAQ scales.

### Methods

#### Participants

The sample consisted of 390 participants (21% undergraduates, 16.7% vocational college students, 25.1% soldiers, 34.6% adults from the community, and 2.6% other; 38.5% females and 61.5% males). The mean age was 25.2 years (*SD* = 8.91) for the entire sample, 20.34 (*SD* = 1.27) for the undergraduates, 18.54 (*SD* = 1.05) for the vocational college students, 21.90 (*SD* = 3.38) for the soldiers, and 33.93 (*SD* = 9.94) for the adults from the community. Thirty-seven percent of the participants (adults from the community and other) filled the questionnaire online and completed 100% of the items and measures. Students and soldiers, recruited from universities, vocational colleges, and military bases in Chongqing and Chengdu, completed the pen-and-paper version in moderately sized groups. Most of them completed 100% of the items and measures. We used data from the participants who completed at least 95% of the items.

#### Procedure

All participants provided their written informed consent. Subsequently, they were asked to complete the questionnaires, including the final PCSAQ and three well-established mental-health-related measures assessing sense of control, happiness, and mood and anxiety symptoms. We used these three measures because these variables reflect psychological health from different angles, and we expected substantial correlations between them and the PCSAQ. To investigate the temporal stability of the PCSAQ, we re-administered it via email 1 week later. However, the average time interval between the two administrations was 10 days, because of the actual time when we received the responses. In the retest session, participants were also given a further mental-health-related measure exploring sense of security. Of the 390 participants, 209 (54%) completed the PCSAQ in the second round.

#### Measures

##### Shapiro control inventory (SCI; Shapiro, 1994)

The SCI is a test that provides a multi-faceted, multi-dimensional control profile of an individual. It can be applied to both psychiatric and non-clinical populations between the ages of 14 and 88 years. This inventory has good reported reliability and validity (Shapiro, 1994). It has nine subscales: General Domain sense of control (3 scales), Modes of Control (4 scales), Domain Specific Sense of Control, and Overall Desire of Control. Items are rated on a 6-point Likert-type scale, with scores ranging from 1 (never) to 6 (very often). *The General Domain Sense of Control Scale (GDSCS)* was adopted for use in the current study to measure a person's view that she/he has control, as well as the belief that she/he can gain control if desired. It encompasses two specific scales: Positive Sense of Control (11 items) and Negative Sense of Control (5 items). The overall sense of control score equals the sum of the positive score plus the reversed negative score. In this study, Cronbach's alpha for the overall sense of control, positive sense of control, and negative sense of control scales were 0.85, 0.89, and 0.84, respectively.

##### Short-form version of the oxford happiness questionnaire (OHQ short scale; Hills and Argyle, 2002)

The OHQ short scale is an 8-item self-report measure of personal happiness. Items are rated on a 6-point Likert-type scale, with scores ranging from 1 (completely disagree) to 6 (completely agree). The short version has good reported validity, which is similar to that of the full 29-item version (Hills and Argyle, 2002). In this study, the Cronbach's alpha for the OHQ short scale was 0.78.

##### Short adaptation of the mood and anxiety symptoms questionnaire (MASQ-D30; Wardenaar et al., 2010)

The original MASQ is a 90-item self-report measure designed to assess the corresponding dimension of the tripartite model of anxiety and depression by Clark and Watson (1991). It was found to have acceptable psychometric properties (Watson et al., 1995). Items are rated on a 5-point Likert-type scale, with scores ranging from 1 (not at all) to 5 (extremely). The MASQ-D30 (Wardenaar et al., 2010) is more suitable for large-scale psychopathology research and its good reliability and validity have been verified. It has three subscales: General Distress (10 items), Anhedonic Depression (10 items), and Anxious Arousal (10 items). In this study, Cronbach's alpha was 0.90, 0.83, 0.86, and 0.82 for the overall scale and the three subscales, respectively.

##### Security-insecurity inventory (S-I; Maslow et al., 1945)

Security is defined as one of the most important determinants of mental health. Based on clinical experiences and Maslow's hierarchy of needs theory, Maslow et al. (1945) developed the S-I, which consists of 75 items and was shown to have good reliability and validity. For each item, there are three choices: yes, no, and not sure. If a response is in line with the scoring key, a score of 0 is earned; otherwise, a score of 1 is earned. The higher the total, the higher the level of insecurity. In this study, Cronbach's alpha for the S-I was 0.92.

#### Data analysis

For each item of the PCSAQ, the score on experience was used for the confirmatory factor analysis (CFA) and reliability analyses. To validate the factor structure from EFA, CFA analyses were conducted with Mplus 7.0 using the mean and variance adjusted weighted least squares (WLSMV) estimation procedure. The WLSMV is a robust estimator that can be used with categorical or ordered indicators since it does not assume normally distributed variables. Thus, it may provide the best option for modeling categorical or ordered data in structural equation modeling framework (Brown, 2006).

Model fit was examined using three commonly used fit indices: the root mean square error of approximation (RMSEA), comparative fit index (CFI), and Tucker-Lewis index (TLI). Hu and Bentler (1999) suggested that CFI and TLI values greater than 0.95 are suggestive of a good fit to the data. RMSEA score of less than 0.05 is suggestive of a good fit to the data. However, these fit indices may be too strict and can be questioned in terms of both practical and substantive significance (Marsh et al., 2004; Hopwood and Donnellan, 2010). Therefore, based on recommendations by West et al. (2012), we adopted CFI and TLI of 0.90 or above and RMSEA of 0.08 or below as indicative of acceptable model fit. Internal reliability was examined using Cronbach's alpha, and the temporal stability of the PCSAQ was examined using the Pearson correlation coefficient.

Regarding validity, we conducted analyses of construct and concurrent validity. For construct validity, we performed analysis of group differences since these groups were expected to have different social experiences, which might result in differences in their mean scores. Specifically, we used four groups of individuals (undergraduates, vocational college students, soldiers, and adults from the community). We choose these four groups because there were similarities and differences among them in real-life situations. Both soldiers and undergraduates live in a relatively uniform environment. In comparison, adults from the community usually have more complex living environments companied by more stress. In China, vocational college students are, on average, inferior to undergraduates with respect to academic record, family environment, and school atmosphere. Consequently, we expected groups living in more complex or negative environments (adults from the community and vocational college students) to have higher scores on the PCSAQ total score. Regarding age, the preliminary analyses of demographic variables in our sample showed that adults from the community were significantly older than the other three groups; soldiers were significantly older than vocational college students; and undergraduates were similar to soldiers and vocational college students. To better explore differences in perceived chronic social adversity due to experiences, we also used age as a covariate.

Using analysis of variance (ANOVA), we examined group mean differences with respect to the total PCSAQ score on experience (which was calculated by summing up the scores on experience for the final 28 items; the PCSAQ-E) and the total PCSAQ score on disturbance (which was calculated by summing up the scores on the disturbance for the final 28 items; the PCSAQ-D). Based on the final version, possible PCSAQ-E total score ranged from 28 to 140. For PCSAQ-D, possible total score ranged from 28 to 112. It should be noted that PCSAQ-E and PCSAQ-D are two aspects of perceived chronic social adversity. We expect that people who experienced more perceived chronic social adversity would feel more disturbed. Therefore, we used these two aspects of PCSAQ in the analysis, although these two aspects of PCSAQ are probably highly correlated.

For concurrent validity, we examined the relationships between the PCSAQ scores (PCSAQ-E and PCSAQ-D) and general domain sense of control, happiness, insecurity, and mood and anxiety symptoms via correlation analyses.

All the analyses mentioned above were performed using SPSS 20.0 except for CFA.

### Results

#### Factor structure

**Figure 1:**
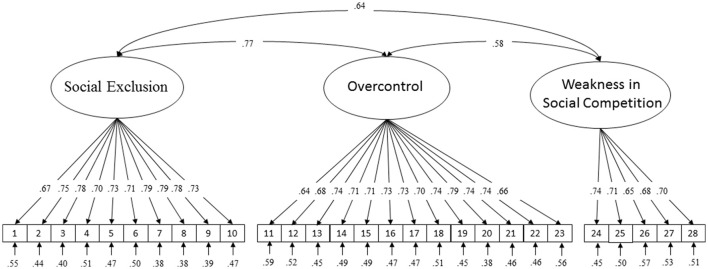
Standardized factor loadings, correlations between factors, and measurement error terms for the three-factor PCSAQ model.

The three-factor model is presented in Figure 1. The model fit the data well: CFI = 0.97, TLI = 0.97, and RMSEA = 0.04. Synthesizing each index, as well as the specificity of the PCSAQ as mentioned above, we believed that the three-factor model had a good fit.

#### Internal reliability and temporal stability

Cronbach's alpha for the overall PCSAQ was 0.92, and 0.88, 0.87, and 0.70 for the OC, SE, and WISC, respectively. The temporal stability of the PCSAQ during an average 10-day interval was 0.78 for the overall PCSAQ, and 0.78, 0.75, and 0.69 for the OC, SE, and WISC, respectively.

#### Construct validity: between-group comparison of PCSAQ scores

Four groups (undergraduates, vocational college students, soldiers, and adults from the community) were compared with respect to PCSAQ-E and PCSAQ-D. First, the simple univariate ANOVA showed that the main effect of group was significant for both the PCSAQ-E and the PCSAQ-D scores. Descriptive statistics and ANOVA results are presented in Table 3. *Post-hoc* comparisons showed that the score of vocational college students was significantly higher than that of the other three groups on both the PCSAQ-E (undergraduates: *M*_*D*_ = 9.12, *p* < 0.01; soldiers: *M*_*D*_ = 7.70, *p* < 0.01; adults from the community: *M*_*D*_ = 6.19, *p* < 0.01) and the PCSAQ-D (undergraduates: *M*_*D*_ = 6.56, *p* < 0.01; soldiers: *M*_*D*_ = 6.53, *p* < 0.01; adults from the community: *M*_*D*_ = 3.96, *p* < 0.05) (*M*_*D*_ = mean difference). There were no significant differences among undergraduates, soldiers, and adults from the community.

**Table 3:** Comparisons among four age groups on the PCSAQ.

		**Groups**				**Model 1**	**Model 2**	
		**Undergraduates**	**Soldiers**	**Vocational college students**	**Adults from the community**	**Group *F***	**Group *F***	**Age *F***
PCSAQ-E	*M*	38.84	40.25	47.95	41.76	7.42^**^	7.78^**^	4.91^*^
	*(SD)*	(8.65)	(12.99)	(13.05)	(13.12)			
	*n*	79	95	64	135			
PCSAQ-D	*M*	38.00	38.02	44.56	40.59	6.08^**^	7.10^**^	5.04^*^
	*(SD)*	(8.29)	(10.49)	(10.58)	(11.62)			
	*n*	77	93	63	135			

* p < 0.05;

** *p < 0.001; PCSAQ-E, the total PCSAQ experiences score; PCSAQ-D, the total PCSAQ disturbance score; Model 1, the results of simple ANOVA; Model 2, the results of univariate ANOVA with age as a covariate*.

Second, because the preliminary analyses of demographic variables showed significant difference among four groups, we conducted a univariate ANOVA with age as a covariate. We used age as a covariate because we wanted to explore differences in perceived chronic social adversity due to experiences in various environments rather than just age. The results showed that the main effect of group was still significant for both the PCSAQ-E and the PCSAQ-D after controlling for age. ANCOVA results are also presented in Table 3. Pairwise comparisons showed that the score of vocational college students was still higher than that of undergraduates and soldiers on both the PCSAQ-E (undergraduates: *M*_*D*_ = 8.72, *p* < 0.01; soldiers: *M*_*D*_ = 6.96, *p* < 0.01) and PCSAQ-D (undergraduates: *M*_*D*_ = 6.20, *p* < 0.01; soldiers: *M*_*D*_ = 5.89, *p* < 0.01), but did not differ from that of adults from the community. Additionally, the score of adults from the community was significantly higher than that of undergraduates and soldiers too on both the PCSAQ-E (undergraduates: *M*_*D*_ = 5.98, *p* < 0.01; soldiers: *M*_*D*_ = 4.22, *p* < 0.05) and PCSAQ-D (undergraduates: *M*_*D*_ = 5.24, *p* < 0.01; soldiers: *M*_*D*_ = 4.92, *p* < 0.01). There were no significant differences between undergraduates and soldiers. Therefore, after controlling for the effects of age, the results indicate that undergraduates experienced less perceived chronic social adversity and less disturbance caused by perceived chronic social adversity than did adults from the community.

#### Concurrent validity: relationship between the PCSAQ and criterion variables

Correlation analyses showed that both the PCSAQ-E and the PCSAQ-D were significantly related to sense of control (*r* = −0.44, *p* < 0.001; *r* = −0.47, *p* < 0.001), happiness (*r* = −0.47, *p* < 0.001; *r* = −0.48, *p* < 0.001), insecurity (*r* = 0.52, *p* < 0.001; *r* = 0.56, *p* < 0.001), and mood and anxiety symptoms (*r* = 0.56, *p* < 0.001; *r* = 0.58, *p* < 0.001), suggesting a good concurrent validity of the PCSAQ.

## Study 3: PCSAQ validation in a clinical sample

To further examine the concurrent validity of the PCSAQ, we explored the relationships between the PCSAQ and the SCI, OHQ, and MASQ-D30 in a clinical sample. Additionally, because we suspected that perceived chronic social adversity could be personally perceived as potentially overwhelming and related to traumatic reactions, we also explored the relationship between perceived chronic social adversity and PTSD symptoms as defined by the DSM-5 and expected a significant relationship between them.

### Methods

#### Participants

Our clinical sample (*n* = 86; 66.3% females and 33.7% males) included both outpatients and inpatients who sought treatment within the department of psychosomatic medicine at two local hospitals. The mean age was 34.64 (*SD* = 12.11) for the entire sample, 33.98 (*SD* = 12.18) for females, and 35.90 (*SD* = 12.07) for males. Of the 86 participants, 24.4% were diagnosed with anxiety disorders, 22.1% as having an anxious state, 16.3% with depressive disorder, and 7.0% as having a depressive state. Additionally, 11.6% of the participants were diagnosed with insomnia, and 18.5% with other conditions (including bipolar disorder, somatoform disorder, PTSD, and obsessive-compulsive disorder). All these diagnoses were made by psychiatrists based on DSM-5 (American Psychiatric Association, 2013).

#### Procedure

All participants voluntarily completed all the questionnaires online. Four participants did not complete some of the required responses on the questionnaire assessing Trauma History Screen (THS; Carlson et al., 2011); however, since all participants completed at least 95% of the items, their data were included in the analyses.

#### Measures

##### Trauma history screen (THS; Carlson et al., 2011)

The THS was developed as very brief and easy-to-complete self-report measure of exposure to high magnitude stressor (HMS) events and persisting posttraumatic distress (PPD) events. HMS events are regarded as sudden events found to cause extreme distress in most of those who are exposed. PPD events are those associated with significant subjective distress lasting longer than a month. The THS total includes 14 types of trauma, of which Events A to L conform to the trauma definition in the DSM-5. The THS was shown to have good to excellent temporal stability for items and trauma types, and excellent stability for overall HMS and PPD scores. Among five samples, the relationship of HMS scores with PTSD symptoms (measured with the PTSD Checklist-Civilian Version) was 0.22–0.41, and that of PPD scores with PTSD symptoms was 0.25–0.38. The THS assessed the frequency of HMS and PPD events and provided detailed information about PPD events.

##### PTSD checklist for DSM-5 (PCL-5; Weathers et al., 2013)

The PCL-5 is a 20-item self-report measure that assesses the four DSM-5 PTSD symptom clusters: intrusion symptoms, avoidance behavior, negative alterations in cognitions and mood, and alterations in arousal and reactivity. Each symptom is rated on a 5-point Likert-type scale, with scores ranging from 0 (not at all) to 4 (extremely). The PCL for the DSM-IV has three versions: PCL-M (military), PCL-C (civilian), and PCL-S (specific). The PCL-5 is very similar to the PCL-S. The PCL has been shown to have excellent diagnostic efficiency. In this study, Cronbach's alpha was 0.95, 0.91, 0.84, 0.89, and 0.81, respectively, for the overall checklist and the four clusters.

Additionally, the three measures used in Study 2 were also employed in the present study. Regarding the SCI, Cronbach's alpha for the overall sense of control, positive sense of control, and negative sense of control scales in study three was 0.85, 0.90, and 0.79, respectively. Cronbach's alpha for the OHQ short scale was 0.75. Regarding the MASQ-D30, Cronbach's alpha was 0.93, 0.89, 0.91, and 0.88 for the overall scale and the three subscales (General Distress, Anhedonic Depression, and Anxious Arousal), respectively.

#### Data analysis

The internal reliability of the PCSAQ was estimated based on the score on experience for each item using Cronbach's alpha. For concurrent validity, we examined the relationships between the PCSAQ scores (PCSAQ-E and PCSAQ-D) and general domain sense of control, happiness, and mood and anxiety symptoms via correlation analyses. Then we used hierarchical linear regression to examine the additional contribution of PCSAQ (as assessed by PCSAQ-E or PCSAQ-D) to PTSD beyond that by sense of control and by trauma as assessed using the THS (Carlson et al., 2011). Because PPD events were included in the HMS events, we only used HMS events as the indicator of other traumatic events. All these analyses were performed using SPSS 20.0.

### Results

#### Internal reliability

Cronbach's alpha was 0.93 for the PCSAQ (PCSAQ-E) in this clinical sample, and 0.92, 0.87, and 0.65 for the OC, SE, and WISC, respectively.

#### PCSAQ-E and PCSAQ-D in the clinical sample

Regarding scores on experience, the mean values for the clinical sample on the PCSAQ-E, SE, OC, and WISC were 48.77 (*SD* = 17.19), 17.41 (*SD* = 6.92), 24.67 (*SD* = 11.06), and 6.69 (*SD* = 2.53), respectively; for disturbance, the mean scores on the PCSAQ-E, SE, OC, and WISC were 46.27 (*SD* = 14.57), 16.90 (*SD* = 6.10), 22.71 (*SD* = 9.26), and 6.65 (*SD* = 2.39), respectively.

#### Concurrent validity

Correlation analyses showed that both the PCSAQ-E and the PCSAQ-D were significantly related to sense of control (*r* = −0.44, *p* < 0.001; *r* = −0.49, *p* < 0.001), happiness (*r* = −0.31, *p* < 0.001; *r* = −0.38, *p* < 0.001), and mood and anxiety symptoms (*r* = 0.54, *p* < 0.001; *r* = 0.53, *p* < 0.001), providing evidence for concurrent validity of the PCSAQ in this clinical sample.

#### Perceived chronic social adversity as a predictor of PTSD severity

As expected, the scores for HMS events and sense of control were significantly correlated with PTSD scores (Table 4). Preliminary analyses showed that HMS was skewed due to extreme outliers. This was probably because some participants who were exposed to repeated stressors had very high scores on HMS events. Consistent with Carlson et al. (2011), we used 95th percentile Winsorization to transform data to reduce the distortion of statistical values due to extreme outliers.

**Table 4:** Correlation among the PCSAQ, HMS, sense of control, and PTSD (*N* = 82).

	**PCSAQ-E**	**PCSAQ-D**	**HMS events**	**Sense of control**	**PTSD**
PCSAQ-E	1				
PCSAQ-D	0.92	1			
HMS events	0.45	0.44	1		
Sense of control	−0.44	−0.50	−0.10	1	
PTSD	0.68	0.65	0.48	−0.43	1

Two hierarchical regression analyses were performed to examine the relationship between perceived chronic social adversity (predictor: PCSAQ-E or PCSAQ-D, separately) and PTSD severity (dependent variable). The results are presented in Table 5 and showed that perceived chronic social adversity significantly predicted PTSD beyond that of HMS events and sense of control. The findings were consistent with our expectation.

**Table 5:** Hierarchical regression analysis for predicting PTSD severity (*N* = 82).

	**Predictor variable**	***B***	**ß**	***R*^2^**	**Δ*R*^2^**	***F***	**Δ*F***
1	Step 1			0.38		23.77^**^	
	HMS events	1.47	0.44^**^				
	Sense of control	−0.56	−0.39^**^				
	Step 2			0.53	0.15	29.30^**^	25.57^**^
	HMS events	0.79	0.24^*^				
	Sense of control	−0.27	−0.19^*^				
	PCSAQ-E	0.50	0.49^**^				
2	Step 1			0.38		23.77^**^	
	HMS events	1.47	0.44^**^				
	Sense of control	−0.56	−0.39^**^				
	Step 2			0.49	0.12	25.42^**^	18.30^**^
	HMS events	0.88	0.26^**^				
	Sense of control	−0.26	−0.18				
	PCSAQ-D	0.53	0.45^**^				

* p < 0.05;

** *p < 0.001; ß is standardized regression coefficient; HMS events, high magnitude stressor events*.

### Discussion

The main purpose of this paper was to develop a conceptually sound and psychometrically robust measure of perceived chronic social adversity, which fills a gap in stress measurement. According to McCann et al. (1988), important universal life themes include safety, trust, power, esteem, and intimacy. Events that directly threaten safety have been systematically assessed in the literature; however, to our knowledge, this is the first study to systematically measure events related to trust, power, esteem, and intimacy that may be perceived as adversity. The cumulative effects of these social events may be less intense but might indeed be personally perceived as potentially overwhelming and be linked to traumatic reactions.

The results of the item response to PCSAQ-E indicated that few individuals (between 5 and 15%) reported moderate to complete agreement across most experiences. Similarly, the data from the PCSAQ-D indicated that few individuals (between 5 and 15%) reported obvious or extreme disturbance. This response pattern was expected, given the nature of what we were assessing, because we expected that only minority of individuals perceived these events as chronic social adversity and might be at higher risk of difficulty, and thus, the number of these respondents should relatively be small.

The three studies in this paper provide evidence for the reliability and construct and concurrent validity of the PCSAQ. The EFA yielded three factors that are in accord with our theoretical expectations. The factor loadings of all final 28 items are higher than 0.32, with 75% of them being greater than 0.50. The three-factor structure of the PCSAQ was further validated with a separate sample using CFA. Reliability analysis showed that the PCSAQ had good internal reliability and temporal stability during an average of 10 days. The concurrent validity of the PCSAQ was supported using a non-clinical sample and a clinical sample, respectively.

With respect to factor structure of PCSAQ, the inter-factor correlations ranged from 0.58 to 0.77. Given the nature of these events, we expected them to be correlated. For example, although social exclusion and overcontrol represent two different patterns of negative treatment, individuals who are excluded or overly controlled may experience a certain degree of loss of autonomy; that is, they cannot be freely attached in the relationship they wish for, or can hardly obtain true intimacy or resources in their existing relationship. Thus, correlation between seemed reasonable.

With respect to group differences, we compared four groups of participants with similarities and differences in real-life situations on the PCSAQ in the study 2. Interestingly, the comparison of the PCSAQ-E and the PCSAQ-D scores between these four groups showed that when not controlling for age, significant differences only existed between vocational college students and the other three groups (undergraduates, soldiers, and adults from the community). However, when age was controlled for as a covariate, the scores of vocational college students were still significantly higher than those of undergraduates and soldiers. Besides, the scores of adults from the community were saliently higher than those of undergraduates and soldiers. These results suggest that the environment may have a stronger effect than does age on perceived chronic social adversity. Moreover, becoming older does not necessarily imply that an individual will experience more perceived chronic social adversity. Instead, older age can become a protective factor. A meta-analysis study confirmed that older adults showed a significant information-processing bias toward positive vs. negative information (Reed et al., 2014).

With respect to concurrent validity, we explored the association between the PCSAQ and various mental health-related indexes in both Studies 2 and 3. Perceived chronic social adversity was significantly correlated with various mental-health-related indexes, including sense of control, insecurity, happiness, anxiety and depression symptoms, and PTSD severity. Specifically, perceived chronic social adversity was significantly correlated with PTSD severity when the effects of trauma were controlled for. This may suggest that perceived chronic social stress contributes traumatic reactions beyond what can be expected from trauma. These results are similar to and expanded the findings of Idsoe et al. (2012) and Matthiesen and Einarsen (2004), who demonstrated an association between PTSD and bullying. Bullying is indeed a broad concept reflected by many PCSAQ items. Despite the evidence for the concurrent validity of the PCSAQ derived in our study, it is important to emphasize that measurement validation is an ongoing process and we recognize that both longitudinal and experimental research will be required to ascertain the predictive validity of the PCSAQ.

Although the goal of this research was to assess perceived chronic social adversity, our deepest concern is still to help individuals exposed to perceived chronic social adversity to achieve positive outcomes in the long term. Despite this objective cannot realize in this research, one avenue for future research may involve investigating important health-enhancing factors moderating perceived chronic social adversity and the various reactions to it. Researches focusing on positive adaptation to trauma or post-traumatic growth have found many important effective factors, such as meaning making (e.g., Mols et al., 2009; Morris and Shakespeare-Finch, 2011), positive coping process (e.g., Thombre et al., 2010), sense of coherence (e.g., Antonovsky, 1987; Veronese and Pepe, 2014), social support (e.g., Nenova et al., 2013), and wisdom (e.g., Linley, 2003). Thus, it may be interesting to consider perceived chronic social adversity in terms of adversarial growth and related factors.

The PCSAQ was developed to measure exposure to three types of stressful/negative events: obvious or obscure social exclusion, overcontrol, and weakness in social competition. Although some existing inventories are related to these three domains, the PCSAQ may have advantages over these in three respects: Firstly, all these inventories focus on a single topic, such as the OESA (Gilman et al., 2013), the PCS-YSR (Barber, 1996), the EPEB (Chen, 2009), the Social Comparison Rating Scale (Allan and Gilbert, 1995), and the SDS (Gilbert and Allan, 1998). Thus, simply combining these inventories together to measure perceived chronic social adversity would involve too many questions and completion would become burdensome for respondents. Secondly, in defining social exclusion, overcontrol, and weakness in social comparison, the PCSAQ adopts a broader definition than do other inventories; thus, it can better and more fully represent perceived chronic social adversity as we aim to measure it. For example, the 11 items of the OESA (Gilman et al., 2013) assess details of ostracism experiences, which can actually be classified as two subtypes of exclusion similar to Item 4 (Always being ignored or left out) and Item 6 (Always being excluded or isolated) of the PCSAQ. However, the PCSAQ also assesses experiences such as deception, suspicion, humiliation, etc., which signal an increased probability of ultimate exclusion. Thirdly, some existing inventories only apply to a particular population (e.g., the OESA), or the events or behaviors described in the items need to be initiated by a specific agent (e.g., the PCS-YSR and EPEB). The PCSAQ was generated to be used with any population with a basic level of literacy and understanding and to explore the superimposed effects of stressful social events regardless of who initiated them. Therefore, the PCSAQ is more generally applicable.

There are some limitations to the current study. First, the participants were recruited using convenience sampling, especially those who voluntarily completed the questionnaire online. Second, the PCSAQ has more global items than do other exposure measures related to interpersonal or personal negative events. The global nature of the PCSAQ may encourage vague answers and influence temporal stability. However, chronic stress itself is difficult to estimate with precision and the PCSAQ focuses on the subjective processing of experience instead of the details of facts. In fact, it is important that clinicians should attempt to appreciate clients' construction of events (Butt and Parton, 2005). Third, our EFA results showed that the three types of events are captured by the measure. One potential problem may be that one cannot know if the EFA captures the three types of events or it captures that the three types of events use three different and confounded question forms (statements which begin with the word “Always” composes one factor, statements that begin with “Somebody” or “Someone” is a second factor, and “It's always” or “I always” is a third factor). In other words, there may be an issue of method variance. Thus, there is a need to find a better way to control the method variance due to wording of items. Fourth, the internal consistency of weakness in social competition was relatively low and needs to be further investigated. Fifth, the PCSAQ could be considered to be a measure of personal characteristics because of the “subjective” nature. However, personal characteristic itself is determined by gene-by-environment interactions and it affects how people perceive events in turn. But we are only interested in how they feel about these events rather than why they feel.

Despite these limitations, our findings support the PCSAQ is a valuable addition to the literation on stress measurement. The PCSAQ provides information about exposure to cumulative social adversity which may likely be perceived as very stressful or even potentially overwhelming. Although these stressors do not involve direct threats to safety or life, they may still be significantly related to psychological suffering. The PCSAQ allows clinicians and researchers to quickly and easily identify critical personal overwhelmed experiences, and may provide a list of key topics for deep conversations.

## Author contributions

Concept and design of the research: JZ, CD. Acquisition, analysis, and interpretation of data: JZ, CD, and CZ. Draft preparation and critical revision: JZ, CD, YT, and DY.

### Conflict of interest statement

The authors declare that the research was conducted in the absence of any commercial or financial relationships that could be construed as a potential conflict of interest.

## Appendix

### Chinese version of the perceived chronic social adversity questionnaire

1. 总是被抛弃（“抛弃”指的是他人在违背或不考虑你意愿的情况下结束与你的人际关系）。
2. 总是遭到不公平对待（“不公平对待”指的是你遭到他人的差别对待，从而导致你利益受损）。
3. 总是遭到冷落或忽视（“忽视”指的是他人当你可有可无、忽略你的存在）。
4. 总是遭到欺骗（“欺骗”指的是他人故意在你面前呈现出虚假的言行，或故意隐瞒部分或全部真相从而导致你的利益受到损害）。
5. 总是遭到排斥或孤立（“排斥或孤立”指的是他人不愿意接纳你、不允许或不欢迎你参与他们的团体、聚会或活动）。
6. 总是遭到拒绝（“拒绝”指的是他人拒绝和你建立你所期望的合作、友好或亲密关系）。
7. 总是遭到嘲笑、戏弄或羞辱（“嘲笑、戏弄或羞辱”指的是他人通过带有讽刺性的语言来取笑你或侮辱你，或者行为上对你进行恶意捉弄）。
8. 总是遭到贬损（“贬损”指的是他人用难听的或带恶意的话语故意降低对你个人的评价，或在你面前贬损你的家庭、种族或宗教）。
9. 总是遭到诽谤或造谣中伤（“诽谤或造谣中伤”指的是他人制造有关于你的谣言并恶意散布，以毁坏你的名誉）。
10. 总是遭到怀疑或不信任（“怀疑或不信任”指的是在你坦诚相待的情况下，他人却对你的言语、行为、动机或感情真实度产生质疑、猜忌）。
11. 某人总是试图改变我的情绪感受和想法。
12. 某人向来不允许我对他（她）的观点或行为提出质疑。
13. 某人总是限制我说话的自由，如限制我谈话的主题、随意打断我的话或转移话题。
14. 某人会在绝大部分事情上强制替我做决定。
15. 某人总是试图改变我做事情的方式。
16. 某人总是将他人或他（她）自己的过错归咎于我。
17. 当某人与我产生争执时，他（她）总是提及我之前所犯的错误。
18. 为了能让我答应他（她）的要求，某人总是故意伤害自己或威胁要伤害自己。
19. 某人总是以伤害我或威胁伤害我的方式迫使我按他（她）的要求行事。
20. 如果我不按某人的要求行事，他（她）就会让我失去我所在乎的东西。
21. 某人总是提醒或暗示我他（她）为我做过很多事或为我做了很多牺牲。
22. 某人总是提醒或暗示我，如果我真的关心他（她），就不会做让他/她担心或烦恼的事情。
23. 某人通过一定方式让我知道他的痛苦是由我带来的，只有我能将他（她）带离困境。
24. 我的成绩或绩效总是落于人后。
25. 我考取（申请）学校或职位总是失败。
26. 我总是被辞退（包括在校期间的职位、工作职位，等）。
27. 我总是难以或无法完成任务。
28. 我参加比赛或参评奖项总是失。
29. 我参加比赛或参评奖项总是是利。
